# Exact soliton, lump, and breather solutions of the (3 + 1)-dimensional Jimbo-Miwa equation via the bilinear neural network method

**DOI:** 10.1038/s41598-026-41485-4

**Published:** 2026-04-04

**Authors:** Hisham H. Hussein, Hosam Mekawey, Ahmed Elsheikh

**Affiliations:** 1 School of Mathematical and Computational Sciences, University of Prince Edward Island (UPEI), hosted by Universities of Canada in Egypt, Cairo Campus, Cairo, 11835 Egypt; 2 Department of Mathematics and Engineering Physics, Faculty of Engineering, Cairo University, Giza, Egypt

**Keywords:** Jimbo-Miwa equation, Neural networks, Lump solutions, Soliton interactions, Hirota bilinear method, Nonlinear waves, Mathematics and computing, Physics

## Abstract

The Jimbo-Miwa (3 + 1)-dimensional nonlinear equation is a well-known high-dimensional extension of integrable models and is widely used to describe nonlinear wave behavior in plasma physics, fluid dynamics, nonlinear optics, and quantum field theory. In this paper, we present a structured symbolic approach based on the Bilinear Neural Network Method (BNNM) to address the complexities associated with solving this equation. The proposed framework combines two complementary approaches: deriving exact soliton solutions through the Hirota bilinear method and developing a neural network-based scheme to obtain analytical approximations of the Jimbo-Miwa dynamics. Within this framework, we introduce several neural network ansatz structures, including 4-2-1, 4-3-1, and deep 4-2-2-1 architectures, which allow for the systematic construction of broad families of exact analytical solutions. Our results include explicit rational lump solutions, periodic breather solutions, and rich hybrid interactions between lumps and solitons. This combined approach not only recovers known solution forms but also provides greater flexibility for exploring solution spaces that are difficult to handle with conventional methods. Furthermore, the neural network framework offers an effective route to approximate analytical solutions of the Jimbo–Miwa model, improving computational efficiency and enhancing predictive capability in application-oriented settings.

## Introduction

Nonlinear partial differential equations (PDEs) have long been recognized as central mathematical models for describing nonlinear phenomena in physics, engineering, and applied sciences. Their analytical solutions, when obtainable, provide powerful tools for understanding wave propagation, interaction dynamics, stability, and the emergence of complex structures in high-dimensional systems. Exact solutions enable efficient modeling and control over nonlinear dynamics, offering insights that go beyond numerical approximations^1–6^.

Among the prominent multidimensional nonlinear PDEs, the Jimbo-Miwa equation (JME) in (3 + 1) dimensions holds a distinguished place. It was originally proposed in the study of Kadomtsev-Petviashvili (KP) hierarchies and integrable systems. The JME governs nonlinear dispersive waves and has attracted increasing attention due to its ability to model diverse high-dimensional nonlinear wave interactions^7–9^.

In plasma physics, the JME equation models the evolution of nonlinear waves in magnetized plasma exhibiting anisotropic dispersion. The additional spatial dimensions account for the oblique propagation of waves relative to the magnetic field. This is a scenario existing in astrophysical plasmas and laboratory fusion devices^10^. Furthermore, in fluid dynamics, the JME describes shallow water waves with weak surface tension, providing a theoretical framework for understanding the genesis of rogue waves^11^. Recent studies by Muhammad et al. and Younas et al. have further highlighted the importance of such high-dimensional models in understanding fractional wave structures and soliton dynamics in complex fluid mediums^12^.

A large body of literature has been devoted to obtaining analytical solutions of the JME and its variants using classical mathematical techniques. The Hirota bilinear method has been widely applied to derive lump and multi-soliton solutions and has been extended through Cole-Hopf transformations to uncover richer structures^13^. Other symbolic and computational approaches, such as Darboux transformations, Lie group analysis, and the inverse scattering method, have also been employed to explore its integrable nature and solution space^14,15^. Nevertheless, while these techniques succeed in constructing certain families of solutions, they often miss more intricate or hybrid forms of localized structures.

In parallel, neural network-based methods have recently emerged as a promising avenue for discovering exact or approximate solutions to nonlinear PDEs. Traditional physics-informed neural networks (PINNs) have been employed to approximate PDE solutions, but they generally provide numerical approximations rather than closed-form analytical expressions^16^. To address this limitation, recent studies have proposed bilinear neural network (BNN) methods that integrate Hirota’s bilinear framework with neural architectures to systematically generate exact solutions^17,18^. For example, the BNN has been applied to the (3 + 1)-dimensional Kairat-X extended equation^17^, to a modified Benney–Luke equation^18^, and to other nonlinear dispersive models, demonstrating its capability in capturing lump, breather, and hybrid structures^17,18^.

Despite this progress, the application of the bilinear neural network method to the Jimbo-Miwa equation has not yet been systematically investigated. This gap is significant because the JME, with its multidimensional and highly nonlinear structure, offers fertile ground for exploring new solution families that may reveal novel wave dynamics and broaden the scope of potential physical applications. Motivated by this, the present work aims to bridge this gap by combining the bilinear neural network framework with the Hirota bilinear method and Cole-Hopf transformations to derive exact analytical solutions for the ($3+1$)-dimensional JME.

The originality of this paper lies in introducing a novel hybrid analytical, neural framework for the systematic study of the (3 + 1)-dimensional JME. Unlike existing approaches, the proposed method integrates a rigorously derived Cole-Hopf transformation that is obtained via truncated Painlevé analysis, with bilinear neural network architectures and the Hirota bilinear method. This integration enables the direct construction of exact analytical solutions while simultaneously uncovering new hybrid wave structures that have not been reported previously. The results demonstrate that neural network-assisted bilinear formulations not only reproduce classical solution families but also significantly expand the solution space of high-dimensional integrable systems.

The main contributions of this paper are summarized as follows:

- We develop a family of bilinear neural network architectures, ranging from simple 4-2-1 models to more expressive 4-4-1 and deep 4-2-2-1 structures, incorporating diverse activation functions such as quadratic, exponential, hyperbolic, and trigonometric forms, to systematically derive exact solutions of the (3 + 1)-dimensional Jimbo–Miwa equation.
- By integrating the Hirota bilinear method with the proposed neural framework, we construct five distinct classes of exact analytical solutions, including lump solutions, lump-soliton composite solutions, lump double-soliton interaction solutions, breather solutions, and novel hybrid mixed-type solutions.
- We present detailed visualizations and qualitative analyses of the obtained solutions, revealing rich nonlinear dynamics characterized by steep localized transitions, asymmetric propagation behaviors, and complex nonlinear wave interaction mechanisms.
- The results validate the Bilinear Neural Network Method (BNNM) as a robust and effective tool for exploring nonlinear wave phenomena in high-dimensional integrable systems and for expanding the solution space beyond classical analytical approaches.

## Jimbo-Miwa equation and the applied method

Complex phenomena in engineering and physics can usually be reduced to PDEs. The study of their properties, especially explicit analytical (soliton) solutions, is crucial for understanding complex behaviors and underlying mechanisms^3,4,9^. Over the years, many effective methods have been developed to construct exact solutions of nonlinear PDEs, including the Hirota bilinear method^13^, Bäcklund-transformation based approaches^19^, Lie symmetry analysis and generalized Kudryashov method^15^, the generalized exp function method^20^, and other integrability based frameworks^7,10,12^.

In this work, we aim to examine the (3 + 1)-dimensional JME given by^9^:

1 $${U}_{xxxy}+3\left({U}_{x}{U}_{xy}+{U}_{y}{U}_{xx}-{U}_{xz}\right)+2{U}_{yt}=0$$

Equation (1) is the well-known Kadomtsev–Petviashvili (KP) hierarchy of integrable systems and used widely to describe some interesting (3 + 1)-dimensional waves in plasma and optics. Up to now, some important research achievements have been developed to deal with Eq. (1)^7–9^.

Now, to obtain the Hirota bilinear form of Eq. (1), we adopt the Cole-Hopf transform as^13^:

2 $$U=2{\left(\mathrm{ln}\left(\psi\right)\right)}_{{x}^{{\prime}}}$$

Taking it into Eq. (1), we can obtain the bilinear form as:

3 $$\left({D}_{x}^{3}{D}_{y}+2{D}_{y}{D}_{t}-3{D}_{x}{D}_{z}\right)\psi\cdot\psi=0$$

Here, the definition of the operators ${D}_{x}^{m}{D}_{\tau}^{n}$ is^11,13^:

4 $$ D_{x}^{m} D_{t}^{n} f \cdot g = \left( {\frac{\partial }{{\partial x}} - \frac{\partial }{{\partial x^{\prime } }}} \right)^{m} \left( {\frac{\partial }{{\partial t}} - \frac{\partial }{{\partial t^{\prime } }}} \right)^{n} f(x,t)g\left( {x^{\prime } ,t^{\prime } } \right)|_{{x = x^{\prime } ,t = t^{\prime } }} $$

To derive the bilinear form of the equation under consideration, we begin by expanding each term using the symmetric bilinear identity. Now to solve our proposed model, we replace both functions $f$ and $g$ with the function $\psi$. Thus, by applying the identity for higher-order bilinear derivatives, the first term, ${D}_{x}^{3}{D}_{y}\psi\cdot\psi$ can be expanded as:

5 $${D}_{x}^{3}{D}_{y}\psi\cdot\psi=2\left({\psi}_{xxxy}\psi-3{\psi}_{xxy}{\psi}_{x}+3{\psi}_{xy}{\psi}_{xx}-{\psi}_{y}{\psi}_{xxx}\right)$$

The second term, $2{D}_{y}{D}_{t}\psi\cdot\psi$, simplifies as:

6 $$2{D}_{y}{D}_{t}\psi\cdot\psi=2\left({\psi}_{yt}\psi-{\psi}_{y}{\psi}_{t}\right)$$

The third term, $-3{D}_{x}{D}_{z}\psi\cdot\psi$, becomes:

7 $$-3{D}_{x}{D}_{z}\psi\cdot\psi=-3\left({\psi}_{xz}\psi-{\psi}_{x}{\psi}_{z}\right)$$

Combining all the terms, we obtain the full expanded form that can be co ncisely expressed as:

8 $$2\left({\psi}_{xxxy}\psi-3{\psi}_{xxy}{\psi}_{x}+3{\psi}_{xy}{\psi}_{xx}-{\psi}_{y}{\psi}_{xxx}\right)+2\left({\psi}_{yt}\psi-{\psi}_{y}{\psi}_{t}\right)-3\left({\psi}_{xz}\psi-{\psi}_{x}{\psi}_{z}\right)=0.$$

## The obtained solutions and discussion

The central methodological contribution of this work is the application of the Bilinear Neural Network Method (BNNM) to the JME. It is essential to clarify that “Neural Network” in this context refers to a symbolic ansatz construction inspired by neural architectures, distinct from data-driven, backpropagation-based models used in machine learning (like PINNs).

### Lump solutions

**Fig. 1:**
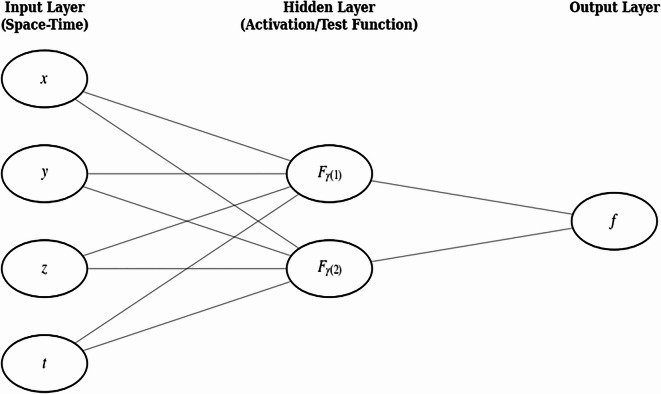
Neural network architecture used for the lumped-solution model. A 4–2–1 feed-forward network with four inputs $(x,y,z,t)$, two hidden neurons with activation functions ${F}_{\gamma\left(1\right)}$ and ${F}_{\gamma\left(2\right)}$,where $\gamma$ stands for 1,2 then a single output $f$, with full connectivity between adjacent layers.

Based on the standard lumped solution form, we construct a 4–2–1 network. The activation functions in layer 2 have the form ${\left({\xi}_{ij}\right)}^{2}$ as specified in Fig. 1.

The specific construction form is as follows:

9 $${\xi}_{11}\left(x,y,z,t\right)={k}_{11}x+{k}_{21}y+{k}_{31}z+{v}_{11}t+{b}_{1}{\xi}_{21}\left(x,y,z,t\right)={k}_{12}x+{k}_{22}y+{k}_{32}z+{v}_{12}t+{b}_{2}$$

10 $${\psi}_{I}\left(x,y,z,t\right)={\omega}_{11}{\left({\xi}_{11}\left(x,y,z,t\right)\right)}^{2}+{\omega}_{21}{\left({\xi}_{21}\left(x,y,z,t\right)\right)}^{2}$$

By substituting with ${\psi}_{I}\left(x,y,z,t\right)$ into Eq. (2), and then into Eq. (1), we obtain the following two sets of solutions,

11 $$SetI-1=\left\{{k}_{21}=\frac{{k}_{22}{k}_{31}}{{k}_{32}},{k}_{12}=-\frac{{k}_{11}{k}_{31}{\omega}_{1}}{{k}_{32}{\omega}_{2}},{v}_{1}=\frac{3{k}_{11}{k}_{32}}{2{k}_{22}},{v}_{2}=-\frac{3{k}_{11}{k}_{31}{\omega}_{11}}{2{k}_{22}{\omega}_{21}}\right\}$$

and

12 $$Set~I - 2=\left\{ \begin{gathered} {v_1}=\frac{{3\left( {{k_{11}}{k_{21}}{k_{31}}{\omega _{11}}+{k_{12}}{k_{22}}{k_{31}}{\omega _{21}} - {k_{12}}{k_{21}}{k_{32}}{\omega _{21}}+{k_{11}}{k_{22}}{k_{32}}{\omega _{21}}} \right)}}{{2\left( {k_{{21}}^{2}{\omega _{11}}+k_{{22}}^{2}{\omega _{21}}} \right)}},~ \hfill \\ {v_2}= - \frac{{3\left( { - {k_{12}}{k_{21}}{k_{31}}{\omega _{11}}+{k_{11}}{k_{22}}{k_{31}}{\omega _{11}} - {k_{11}}{k_{21}}{k_{32}}{\omega _{11}} - {k_{12}}{k_{22}}{k_{32}}{\omega _{21}}} \right)}}{{2\left( {k_{{21}}^{2}{\omega _{11}}+k_{{22}}^{2}{\omega _{21}}} \right)}},~ \hfill \\ {b_1}=\frac{{2\left( {k_{{11}}^{2}{\omega _{11}}+k_{{12}}^{2}{\omega _{21}}} \right)\left( {{k_{11}}{k_{21}}{\omega _{11}}+{k_{12}}{k_{22}}{\omega _{21}}} \right)\left( {k_{{21}}^{2}{\omega _{11}}+k_{{22}}^{2}{\omega _{21}}} \right)}}{{\left( { - {k_{12}}{k_{21}}+{k_{11}}{k_{22}}} \right)\left( {{k_{22}}{k_{31}} - {k_{21}}{k_{32}}} \right){\omega _{11}}{\omega _{21}}}}.~ \hfill \\ \end{gathered} \right\}$$

The general form of the solutions to Eq. (1) corresponds to case I,

13 $${G}_{I-\left(i\right)}\left(x,y,z,t\right)=4\frac{{k}_{11}{\omega}_{11}{\xi}_{11}\left(x,y,z,t\right)+{k}_{21}{\omega}_{21}{\xi}_{21}\left(x,y,z,t\right)}{{\omega}_{11}{\left({\xi}_{11}\left(x,y,z,t\right)\right)}^{2}+{\omega}_{21}{\left({\xi}_{21}\left(x,y,z,t\right)\right)}^{2}}$$

where $i$ stands for the number of the obtained sets of solutions and corresponds for the number of the sets of solutions. Here, ${U}_{I-\left(i\right)}\left(x,y,z,t\right)$ is expressed in terms of two linear arguments, ${\xi}_{11}\left(x,y,z,t\right)$ and ${\xi}_{21}\left(x,y,z,t\right)$, which are combinations of spatial and temporal variables $\left(xandtrespectively\right)$. The numerator of this function grows linearly with respect to these arguments, while the denominator depends quadratically on them. This algebraic structure results in a wave profile characterized by steep localized variations where the denominator approaches small values, and smoother decaying behavior away from these regions.

### Interaction of lump with soliton

To better understand the physical properties of the bilinear equation, we construct a 4-2-1 model to explore the interactions between lump solutions and solitons. The activation functions have been set as ${F}_{1}\left({\xi}_{ij}\right)={\left({\xi}_{ij}\right)}^{2}$ and ${F}_{2}\left({\xi}_{ij}\right)={e}^{{\xi}_{ij}}$, with the same constructed neural network model shown in Fig. 1. The constructed structure is

14 $${\xi}_{12}\left(x,y,z,t\right)={l}_{11}x+{l}_{12}y+{l}_{13}z+{v}_{21}t+{d}_{1}{\xi}_{22}\left(x,y,z,t\right)={l}_{21}x+{l}_{22}y+{l}_{23}z+{v}_{22}t$$

15 $${\psi}_{II}\left(x,y,z,t\right)={\omega}_{12}{\left({\xi}_{12}\left(x,y,z,t\right)\right)}^{2}+{\omega}_{22}{e}^{{\xi}_{22}\left(x,y,z,t\right)}+{\omega}_{32}$$

Substituting Eqs. (14) and (15) into Eqs. (1) and (2), and solving the resulting algebraic system, we obtain the following sets:

16 $$SetII-1=\left\{{l}_{21}=1,{l}_{22}=\frac{3{l}_{23}}{2\left(1+{v}_{22}\right)},{\omega}_{12}=0\right\},$$

17 $$SetII-2=\left\{{l}_{12}={l}_{13}=0,{l}_{21}=1,{l}_{22}=\frac{3{l}_{23}}{2\left(1+{v}_{22}\right)},{v}_{21}={l}_{11}\left(1+{v}_{22}\right)\right\},$$

18 $$SetII-3=\left\{{l}_{21}=1,{l}_{12}=\frac{3{l}_{11}{l}_{23}}{2\left(1+{v}_{22}\right)},{l}_{13}=\frac{{l}_{23}{v}_{21}}{1+{v}_{22}},{l}_{22}=\frac{3{l}_{23}}{2\left(1+{v}_{22}\right)},{\omega}_{12}=0\right\}$$

and

19 $$SetII-4=\left\{{l}_{21}=1,{l}_{13}=\frac{2}{3}{l}_{12}\left(1+{v}_{22}\right),{l}_{22}=\frac{3{l}_{23}}{2\left(1+{v}_{22}\right)},{\omega}_{21}=0,{v}_{21}={l}_{11}\left(1+{v}_{22}\right)\right\}.$$

The general form of the solutions to Eq. (1) corresponds to case II,

20 $${G}_{II-\left(i\right)}\left(x,y,z,t\right)=2\frac{{l}_{11}{\omega}_{12}{\xi}_{12}\left(x,y,z,t\right)+{l}_{21}{\omega}_{22}{e}^{{\xi}_{22}\left(x,y,z,t\right)}}{{\omega}_{12}{\left({\xi}_{12}\left(x,y,z,t\right)\right)}^{2}+{\omega}_{22}{e}^{{\xi}_{22}\left(x,y,z,t\right)}+{\omega}_{32}}$$

The structure of ${G}_{II-\left(i\right)}\left(x,y,z,t\right)$ differs from the previous case due to the inclusion of an exponential dependence in both the numerator and denominator. The function is therefore composed of a linear term in ${\xi}_{12}\left(x,y,z,t\right)$ and an exponential term in ${\xi}_{22}\left(x,y,z,t\right)$, balanced by a denominator that combines quadratic, exponential, and constant contributions. This composition gives rise to richer dynamical behavior, as the exponential term introduces strong asymmetry in the growth and decay of the function across different regions of the $\left(x,t\right)$ plane.

To explore the full range of this function’s behavior, several parameter sets are introduced. Set II–1 simplifies the expression by imposing ${\omega}_{12}=0$, thereby eliminating the quadratic contribution in the denominator and leaving the balance primarily between the exponential growth of ${\xi}_{22}\left(x,y,z,t\right)$​ and the constant term ${\omega}_{32}$​. Set II–2 removes contributions from ${l}_{12}$​ and ${l}_{13}$​ while preserving the exponential term, making the function dominated by the interplay of the exponential excitation and the parameter-controlled velocity term ${v}_{21}$​. Set II–3 restores quadratic contributions through ${l}_{12}$​ and ${l}_{13}$​ but enforces ${\omega}_{12}$, yielding a structure that still emphasizes exponential asymmetry but with additional coupling between spatial and temporal growth rates. Finally, Set II–4 introduces relations between ${l}_{12}$​ and ${l}_{13}$​ while setting ${\omega}_{21}=0$, combining polynomial and exponential contributions in a way that modifies the propagation velocity through the explicit dependence of ${v}_{21}$​ on ${l}_{11}$​ and ${v}_{22}$​.

Overall, the form of ${G}_{II-\left(i\right)}\left(x,y,z,t\right)$ highlights the competition between linear, quadratic, and exponential components, producing solutions that can exhibit steep fronts, asymmetric propagation, and localized growth depending on the parameter set employed. Each of the four parameters tunes the relative importance of these contributions, enabling the function to describe a variety of nonlinear wave behaviors, ranging from simplified exponential resonances to more intricate coupled interactions involving both polynomial and exponential growth terms.

### Interaction of Lump with double solitons

**Fig. 2:**
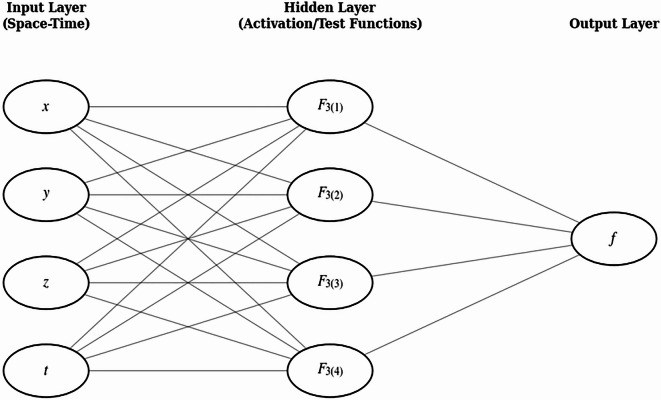
Neural network architecture used for the bilinear-equation study. A 4–4–1 feed-forward network with four inputs $x,y,z,t$, four hidden neurons with activation functions ${F}_{3\left(1\right)},{F}_{3\left(2\right)},{F}_{3\left(3\right)},{F}_{3\left(4\right)}$, and a single output $f$, with full connectivity between adjacent layers.

To examine the bilinear equation’s physical behavior, we build a 4–4–1 network to study interactions between lump solutions and double solitons. The activation functions are set as ${\left({\xi}_{ij}\right)}^{2}$ and ${e}^{{\xi}_{ij}}$.The model structure is shown in Figure 2.

The inputs of layer 1 can be defined as

21 $$\begin{aligned} {\xi _{13}}\left( {x,y,z,t} \right) & ={{\tilde {l}}_{11}}x+{{\tilde {l}}_{12}}y+{{\tilde {l}}_{13}}z+{v_{31}}t+{e_1}{\xi _{23}}\left( {x,y,z,t} \right) \\ ={{\tilde {l}}_{21}}x+{{\tilde {l}}_{22}}y+{{\tilde {l}}_{23}}z+{v_{32}}t+{e_2}{\xi _{33}}\left( {x,y,z,t} \right) \\ ={{\tilde {l}}_{31}}x+{{\tilde {l}}_{32}}y+{{\tilde {l}}_{33}}z+{v_{33}}t+{e_3}{\xi _{43}}\left( {x,y,z,t} \right) \\ ={{\tilde {l}}_{41}}x+{{\tilde {l}}_{42}}y+{{\tilde {l}}_{43}}z+{v_{34}}t+{e_4} \\ \end{aligned} $$

22 $${\psi}_{III}\left(x,y,z,t\right)={\omega}_{13}{\left({\xi}_{13}\left(x,y,z,t\right)\right)}^{2}+{\omega}_{23}{\left({\xi}_{23}\left(x,y,z,t\right)\right)}^{2}+{\omega}_{33}{e}^{{\xi}_{33}\left(x,y,z,t\right)}+{\omega}_{43}{e}^{{\xi}_{43}\left(x,y,z,t\right)}$$

The general form of the resulting solution of case III has the form,

23 $${G}_{III-\left(i\right)}\left(x,y,z,t\right)=2\frac{2{\omega}_{13}{\stackrel{\sim}{l}}_{11}{\xi}_{13}\left(x,y,z,t\right)+2{\omega}_{23}{\stackrel{\sim}{l}}_{21}{\xi}_{23}\left(x,y,z,t\right)+{\omega}_{33}{\stackrel{\sim}{l}}_{31}{e}^{{\xi}_{33}\left(x,y,z,t\right)}+{\omega}_{43}{\stackrel{\sim}{l}}_{41}{e}^{{\xi}_{43}\left(x,y,z,t\right)}}{{\omega}_{13}{\left({\xi}_{13}\left(x,y,z,t\right)\right)}^{2}+{\omega}_{23}{\left({\xi}_{23}\left(x,y,z,t\right)\right)}^{2}+{\omega}_{33}{e}^{{\xi}_{33}\left(x,y,z,t\right)}+{\omega}_{43}{e}^{{\xi}_{43}\left(x,y,z,t\right)}}$$

where the coefficients can be defined as,

24 $$Set~III - 1=\left\{ {{{\tilde {l}}_{13}}=\frac{{{{\tilde {l}}_{11}}{{\tilde {l}}_{23}}}}{{{{\tilde {l}}_{21}}}},{{\tilde {l}}_{22}}=\frac{{{{\tilde {l}}_{12}}{{\tilde {l}}_{21}}}}{{{{\tilde {l}}_{11}}}},{{\tilde {l}}_{31}}=0,~{{\tilde {l}}_{32}}=\frac{{ - 3{{\tilde {l}}_{33}} - 2{{\tilde {l}}_{42}}+3{{\tilde {l}}_{43}}+2{{\tilde {l}}_{42}}{v_{33}} - 2{{\tilde {l}}_{42}}{v_{34}}}}{{2\left( { - 1+{v_{33}} - {v_{34}}} \right)}},{{\tilde {l}}_{41}}=1,{\omega _{31}}= - \frac{{\tilde {l}_{{21}}^{2}{\omega _{32}}}}{{\tilde {l}_{{11}}^{2}}},{v_{31}}=\frac{{{{\tilde {l}}_{11}}{v_{32}}}}{{{{\tilde {l}}_{21}}}},{e_2}=\frac{{{e_1}{{\tilde {l}}_{21}}}}{{{{\tilde {l}}_{11}}}}~} \right\}$$

25 $$Set~III - 2=\left\{ {{{\tilde {l}}_{13}}=\frac{{{{\tilde {l}}_{11}}{{\tilde {l}}_{23}}}}{{{{\tilde {l}}_{21}}}},{{\tilde {l}}_{22}}=\frac{{{{\tilde {l}}_{12}}{{\tilde {l}}_{21}}}}{{{{\tilde {l}}_{11}}}},{{\tilde {l}}_{31}}=1,~{{\tilde {l}}_{32}}=\frac{{3{{\tilde {l}}_{33}}+2{{\tilde {l}}_{42}} - 3{{\tilde {l}}_{43}}+2{{\tilde {l}}_{42}}{v_{33}} - 2{{\tilde {l}}_{42}}{v_{34}}}}{{2\left( {1+{v_{33}} - {v_{34}}} \right)}},{{\tilde {l}}_{41}}=0,{\omega _{31}}= - \frac{{\tilde {l}_{{21}}^{2}{\omega _{32}}}}{{\tilde {l}_{{11}}^{2}}},{v_{31}}=\frac{{{{\tilde {l}}_{11}}{v_{32}}}}{{{{\tilde {l}}_{21}}}},{e_2}=\frac{{{e_1}{{\tilde {l}}_{21}}}}{{{{\tilde {l}}_{11}}}}~} \right\},$$

26 $$Set~III - 3=\left\{ {{{\tilde {l}}_{13}}=\frac{{{{\tilde {l}}_{11}}{{\tilde {l}}_{23}}}}{{{{\tilde {l}}_{21}}}},{{\tilde {l}}_{22}}=\frac{{{{\tilde {l}}_{12}}{{\tilde {l}}_{21}}}}{{{{\tilde {l}}_{11}}}},{{\tilde {l}}_{31}}={{\tilde {l}}_{41}}=1,{{\tilde {l}}_{32}}={{\tilde {l}}_{42}},~{\omega _{31}}= - \frac{{\tilde {l}_{{21}}^{2}{\omega _{32}}}}{{\tilde {l}_{{11}}^{2}}},{v_{31}}=\frac{{{{\tilde {l}}_{11}}{v_{32}}}}{{{{\tilde {l}}_{21}}}},{e_2}=\frac{{{e_1}{{\tilde {l}}_{21}}}}{{{{\tilde {l}}_{11}}}}~} \right\},$$

27 $$Set~III - 4=\left\{ {{{\tilde {l}}_{13}}=\frac{{{{\tilde {l}}_{11}}{{\tilde {l}}_{23}}}}{{{{\tilde {l}}_{21}}}},{{\tilde {l}}_{22}}=\frac{{{{\tilde {l}}_{12}}{{\tilde {l}}_{21}}}}{{{{\tilde {l}}_{11}}}},{l_{31}}= - \frac{{{{32}^{\frac{1}{3}}}\left( {{l_{33}} - {l_{43}}} \right)}}{{{L_0}}}+\frac{{{L_0}}}{{{{62}^{\frac{1}{3}}}{l_{42}}}},~{l_{32}}={l_{41}}=0,{\omega _{31}}= - \frac{{l_{{21}}^{2}{\omega _{32}}}}{{l_{{11}}^{2}}},{v_{31}}=\frac{{{l_{11}}{v_{32}}}}{{{l_{21}}}},{e_2}=\frac{{{e_1}{l_{21}}}}{{{l_{11}}}}~} \right\}$$

and

28 $$Set~III - 5=\left\{ {{l_{13}}=\frac{{{l_{11}}{l_{23}}}}{{{l_{21}}}},{l_{22}}=\frac{{{l_{12}}{l_{21}}}}{{{l_{11}}}},{l_{31}}= - \left( {\frac{{{{32}^{\frac{1}{3}}}\left( {{l_{33}} - {l_{43}}} \right)}}{{{L_2}^{{\frac{1}{3}}}}}} \right)+\frac{1}{{{{62}^{\frac{1}{3}}}{{\tilde {l}}_{42}}}}{L_2}^{{\frac{1}{3}}},~{l_{32}}=0,{l_{41}}=1,{\omega _{31}}= - \frac{{l_{{21}}^{2}{\omega _{32}}}}{{l_{{11}}^{2}}},{v_{31}}=\frac{{{l_{11}}{v_{32}}}}{{{l_{21}}}},{e_2}=\frac{{{e_1}{l_{21}}}}{{{l_{11}}}}~} \right\}$$

where the constants ${L}_{0},{L}_{1},{L}_{2}$ and ${L}_{3}$ can be defined as

$${L}_{0}={\left(-216{l}_{42}^{3}{v}_{33}+216{l}_{42}^{3}{v}_{34}+{L}_{1}\right)}^{\frac{1}{3}},$$

$${L}_{1}=\sqrt{23328{l}_{42}^{3}{\left({l}_{33}-{l}_{43}\right)}^{3}+{\left(-216{l}_{42}^{3}{v}_{33}+216{l}_{42}^{3}{v}_{34}\right)}^{2}},$$

$${L}_{2}=324{l}_{33}{l}_{42}^{2}+216{l}_{42}^{3}-324{l}_{42}^{2}{l}_{43}-216{l}_{42}^{3}{v}_{33}+216{l}_{42}^{3}{v}_{34}+{L}_{3}$$.

and

$${L}_{3}=\sqrt{23328{l}_{42}^{3}{({l}_{33}-{l}_{43})}^{3}+{\left(324{l}_{33}{l}_{42}^{2}+216{l}_{42}^{3}-324{l}_{42}^{2}{l}_{43}-216{l}_{42}^{3}{v}_{33}+216{l}_{42}^{3}{v}_{34}\right)}^{2}}$$.

This function generalizes the previous forms by incorporating both quadratic and exponential contributions in multiple directions simultaneously. The numerator consists of two linear terms with the weighted coefficients ${\omega}_{13}{\stackrel{\sim}{l}}_{11}$ and ${\omega}_{23}{\stackrel{\sim}{l}}_{21}$, as well as two exponential functions with the weighted coefficients ${\omega}_{33}{\stackrel{\sim}{l}}_{31}$ and ${\omega}_{43}{\stackrel{\sim}{l}}_{41}$ that introduce asymmetry and rapid growth in localized regions. The denominator mirrors this structure, containing quadratic suppression terms for $\left({\xi}_{13}\left(x,y,z,t\right),{\xi}_{23}\left(x,y,z,t\right)\right)$ and exponential growth terms for $\left({\xi}_{33}\left(x,y,z,t\right),{\xi}_{43}\left(x,y,z,t\right)\right)$. As a result, the function exhibits a delicate balance between polynomial and exponential influences, leading to a wide spectrum of possible localized structures and propagation patterns.

To explore the richness of this solution, several parameter sets are considered. Set III–1 suppresses the exponential contribution from ${\stackrel{\sim}{l}}_{31}$​ by setting it to zero, while enforcing a specific relation between ${\stackrel{\sim}{l}}_{32},{\stackrel{\sim}{l}}_{42}$ and ${\stackrel{\sim}{l}}_{43}$ and the time coefficients ${v}_{33}$ and ${v}_{43}$​. This configuration highlights the contribution of ${\xi}_{43}\left(x,y,z,t\right)$ while maintaining strong coupling between the linear terms ${\xi}_{13}\left(x,y,z,t\right)$ and ${\xi}_{23}\left(x,y,z,t\right)$​. Set III–2 instead eliminates ${\stackrel{\sim}{l}}_{41}$​, thereby removing the ${\xi}_{43}\left(x,y,z,t\right)$​ exponential while retaining the ${\xi}_{33}\left(x,y,z,t\right)$​ driven contribution. The conditions imposed on ${\stackrel{\sim}{l}}_{32}$​ and related parameters produce an alternative asymmetric structure, balancing growth along different spatial directions. Set III–3 activates both exponential channels $\left({\stackrel{\sim}{l}}_{31}={\stackrel{\sim}{l}}_{41}=1\right)$ and enforces equality ${\stackrel{\sim}{l}}_{32}={\stackrel{\sim}{l}}_{42}$​, resulting in coupled exponential excitations that strongly enhance the nonlinear behavior of the solution. Finally, Set III–4 introduces a more intricate dependence of ${\stackrel{\sim}{l}}_{31}$​ on the remaining parameters, while setting ${\stackrel{\sim}{l}}_{32}={\stackrel{\sim}{l}}_{41}=0$. This construction reduces the effective number of exponential channels while introducing nonlinear scaling relations, thereby generating qualitatively different localized dynamics.

In summary, the form of ${G}_{III-\left(i\right)}\left(x,y,z,t\right)$ reflects a higher-order resonant structure that combines polynomial growth, quadratic suppression, and exponential amplification. The variety of parameter sets allows for tuning the solution from single-channel dominance (linear or exponential) to strongly coupled multi-channel interactions. Physically, this indicates that the function can model complex resonant soliton structures, where multiple wavefronts or localized excitations interact and propagate in a coordinated yet asymmetric fashion. Such configurations are particularly relevant in multi-dimensional nonlinear systems, where the coexistence of linear and exponential modes leads to rich dynamical phenomena.

### Breather solution

Breathing solitons are a type of nonlinear localized wave that exhibit both periodicity and localization, with peaks that fluctuate exponentially. They have numerous important applications across various fields, including the generation of supercontinuum spectra and optical frequency combs, as well as the explanation of turbulent phenomena.

**Fig. 3:**
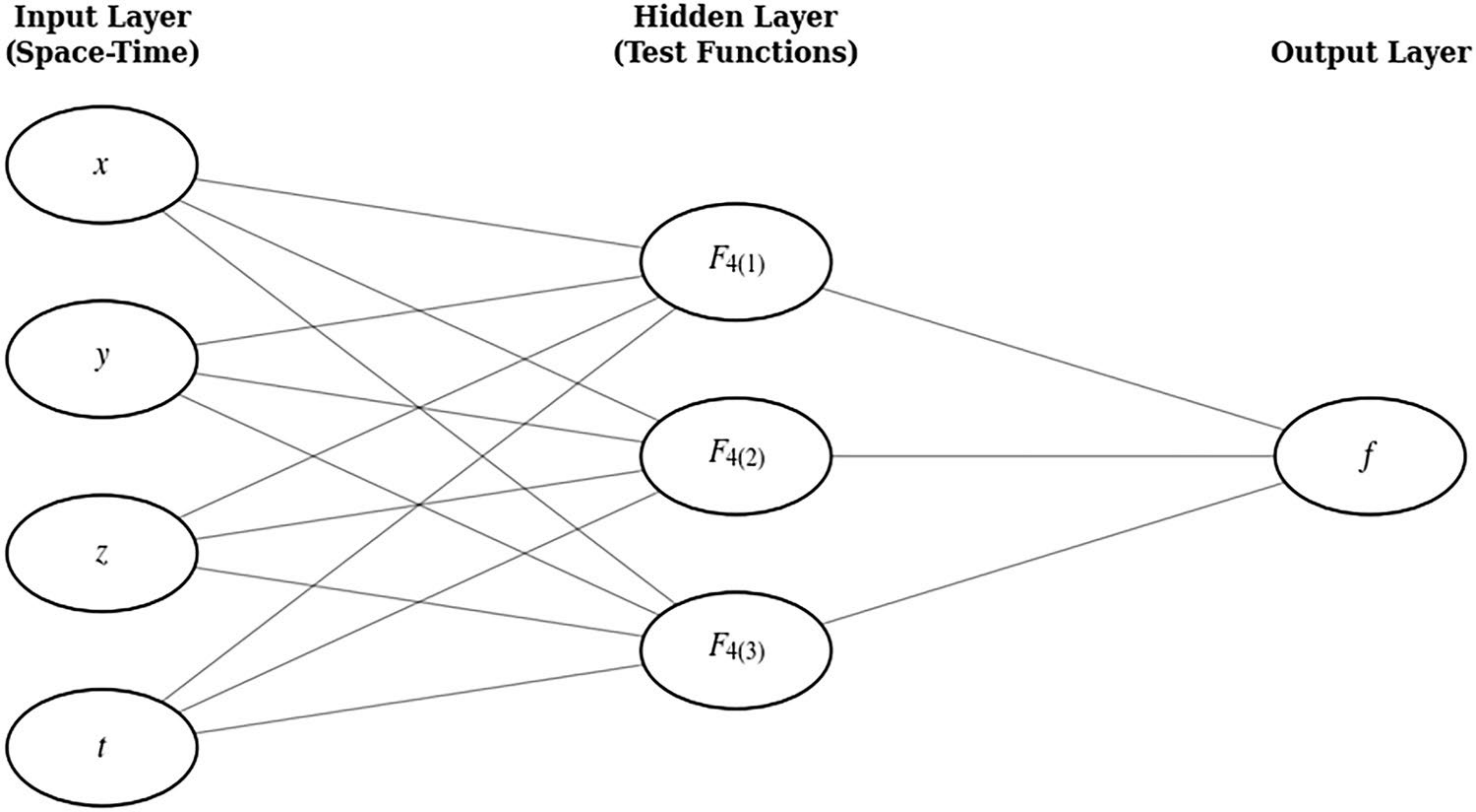
Neural network architecture used for the bilinear-equation study. A 4–3–1 feed-forward network with four inputs $x,y,z,t$, four hidden neurons with activation functions ${F}_{4\left(1\right)},{F}_{4\left(2\right)},$ ${F}_{4\left(3\right)}$ and a single output $f$, with full connectivity between adjacent layers.

To obtain the breathing solutions of Eq. (6), we can construct a neural network model as shown in Fig. 3 as constructed in the following form:

29 $${\xi}_{14}\left(x,y,z,t\right)={n}_{11}x+{n}_{12}y+{n}_{13}z+{v}_{41}t+{f}_{1}{\xi}_{24}\left(x,y,z,t\right)={n}_{21}x+{n}_{22}y+{n}_{23}z+{v}_{42}t+{f}_{2}{\xi}_{34}\left(x,y,z,t\right)={n}_{31}x+{n}_{32}y+{n}_{33}z+{v}_{43}t+{f}_{3}$$

30 $${\psi}_{IV}\left(x,y,z,t\right)={\omega}_{14}sinh\left({\xi}_{14}\left(x,y,z,t\right)\right)+{\omega}_{24}{\left({\xi}_{24}\left(x,y,z,t\right)\right)}^{2}+{\omega}_{34}sin\left({\xi}_{34}\left(x,y,z,t\right)\right)$$

The general form of the resulting solution of case IV has the form,

31 $${G}_{IV-\left(i\right)}\left(x,y,z,t\right)=2\frac{{n}_{11}{\omega}_{14}cosh\left({\xi}_{14}\left(x,y,z,t\right)\right)+2{n}_{21}{\omega}_{24}{\xi}_{24}\left(x,y,z,t\right)+{n}_{31}{\omega}_{34}cos\left({\xi}_{34}\left(x,y,z,t\right)\right)}{{\omega}_{14}sinh\left({\xi}_{14}\left(x,y,z,t\right)\right)+{\omega}_{24}{\left({\xi}_{24}\left(x,y,z,t\right)\right)}^{2}+{\omega}_{34}sin\left({\xi}_{34}\left(x,y,z,t\right)\right)}$$

The form-based solutions can be classified according to the corresponding sets of coefficient values,

32 $$SetIV-1=\left\{{n}_{22}={n}_{23}={n}_{31}={\omega}_{41}={v}_{43}=0,{n}_{33}=\frac{2{n}_{32}{v}_{42}}{3{n}_{21}}\right\},$$

33 $$SetIV-2=\left\{{n}_{12}={n}_{13}={n}_{31}={\omega}_{42}={v}_{43}=0,{n}_{33}=\frac{2{n}_{32}({n}_{11}^{3}+{v}_{41})}{3{n}_{11}}\right\},$$

34 $$SetIV-3=\left\{{n}_{12}={n}_{13}={n}_{22}={n}_{23}={n}_{31}={v}_{43}=0,{n}_{33}=\frac{2{n}_{32}\left({n}_{11}^{3}+{v}_{41}\right)}{3{n}_{11}},{v}_{42}=\frac{{n}_{21}\left({n}_{11}^{3}+{v}_{41}\right)}{{n}_{11}}\right\}$$

and

35 $$SetIV-4=\left\{{n}_{12}={n}_{13}={n}_{21}={n}_{31}={v}_{42}={v}_{43}=0,{n}_{23}=\frac{2\left({n}_{11}^{3}{n}_{22}+{n}_{22}{v}_{41}\right)}{3{n}_{11}},{n}_{33}=\frac{2{n}_{32}({n}_{11}^{3}+{v}_{41})}{3{n}_{11}}\right\}$$

This solution function introduces hyperbolic and trigonometric components alongside linear and quadratic terms, which distinguishes it from the earlier purely polynomial-exponential forms. In the numerator, the contributions of a hyperbolic cosine $cosh\left({\xi}_{14}\right)$, a linear dependence on ${\xi}_{24}$, and a trigonometric cosine $cos\left({\xi}_{34}\right)$ combine to produce competing localized excitations with oscillatory and exponentially growing behavior. The denominator balances these effects with hyperbolic sine $\left(sinh\left({\xi}_{14}\right)\right)$, a quadratic contribution in ${\xi}_{24}$, and a sinusoidal term $sin\left({\xi}_{34}\right)$. The interplay of hyperbolic, trigonometric, and polynomial contributions leads to a rich dynamical structure characterized by alternating growth, decay, and oscillatory modulation.

On the other hand, several parameter sets are considered to extract specific regimes of this function. Set IV-1 eliminates contributions from ${n}_{22},{n}_{23},{n}_{31},{v}_{43}$, and ${\omega}_{41}$, simplifying the dependence on ${\xi}_{24}$ while enforcing a constraint on ${n}_{33}$ through ${n}_{32}$ and ${v}_{42}$. This form enhances the role of the hyperbolic channel and quadratic term, leading to an interplay between steep localized growth and parabolic suppression. Set IV-2 suppresses the contributions from ${\xi}_{14}$ in the transverse directions by setting ${n}_{12}={n}_{13}=0$ and removes the trigonometric channel by imposing ${n}_{31}=0$. The resulting structure highlights hyperbolic growth, with ${n}_{33}$ depending explicitly on both the cubic term ${n}_{11}^{3}$ and the velocity parameter ${v}_{41}$, which modifies the propagation properties of the solution. Set IV-3 further restricts the function by eliminating ${n}_{12},{n}_{13},{n}_{22},{n}_{23},{n}_{31}$, and ${v}_{43}$, while introducing a velocity coupling condition ${v}_{42}=\frac{{n}_{21}\left({n}_{11}^{3}+{v}_{41}\right)}{{n}_{11}}$. This configuration produces a highly constrained system where hyperbolic and quadratic contributions dominate, yielding a more asymmetric propagation profile. Finally, Set IV-4 suppresses most of the transverse contributions by setting ${n}_{12}={n}_{13}={n}_{21}={n}_{31}={v}_{42}={v}_{43}=0$, while introducing explicit parameter dependencies for ${n}_{23}$ and ${n}_{33}$. This construction creates a balance between cubic-velocity coupling and hyperbolic growth, with reduced oscillatory influence from the trigonometric channel.

Overall, the function ${G}_{IV-\left(i\right)}\left(x,y,z,t\right)$ (Eq. (31)) represents a resonant structure where hyperbolic, polynomial, and trigonometric modes coexist. The parameter sets act as tuning controls that enhance or suppress specific components, leading to a range of nonlinear behaviors such as exponential localization, oscillatory modulation, and parabolic suppression. Depending on the imposed constraints, these effects can appear along different spatial or temporal directions. Physically, such solutions model mixed resonant excitations where oscillatory and exponentially growing modes interact, giving rise to hybrid soliton- or breather-like dynamics in nonlinear media.

### “4-2-2-1” neural network model

**Fig. 4:**
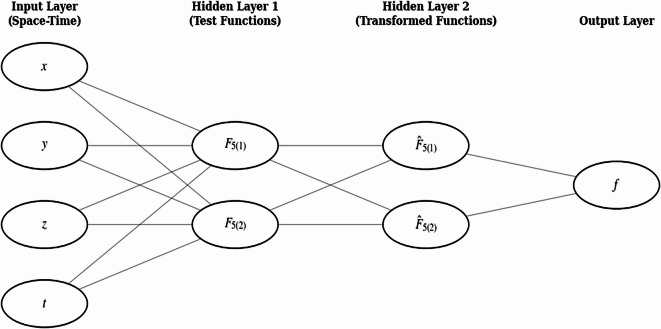
Neural network architecture used for the bilinear-equation study. The model is a 4–2–2–1 feed-forward network with four inputs $\left(x,y,z,t\right)$, two hidden layers with two neurons each, with the activation functions ${F}_{5\left(1\right)},{F}_{5\left(2\right)},{\widehat{F}}_{5\left(1\right)},{\widehat{F}}_{5\left(2\right)}$ and a single output $f$. Each layer is fully connected to the next, with activation functions applied in the hidden layers.

To better investigate the physical significance of the bilinear equation, an implicit layer is constructed using specific functional forms $sin\left({\delta}_{1}\right)$, $cos\left({\delta}_{2}\right),{e}^{{\delta}_{3}}$ and ${\delta}_{4}^{2}$, which serve as the foundation for developing a neural network model, as illustrated in Fig. 4.

$${\xi}_{15}\left(x,y,z,t\right)={o}_{11}x+{o}_{12}y+{o}_{13}z+{v}_{51}t+{g}_{1}$$

36 $${\xi}_{25}\left(x,y,z,t\right)={o}_{21}x+{o}_{22}y+{o}_{23}z+{v}_{52}t+{g}_{2}$$

$${\delta}_{1}\left(x,y,z,t\right)={\eta}_{11}cos\left({\xi}_{15}\left(x,y,z,t\right)\right)+{\eta}_{12}sin\left({\xi}_{25}\left(x,y,z,t\right)\right)$$

37 $${\delta}_{2}\left(x,y,z,t\right)={\eta}_{21}cos\left({\xi}_{25}\left(x,y,z,t\right)\right)+{\eta}_{22}sin\left({\xi}_{15}\left(x,y,z,t\right)\right)$$

38 $${\psi}_{V}\left(x,y,z,t\right)={\omega}_{51}{e}^{\delta1\left(x,y,z,t\right)}+{\omega}_{52}{\left({\delta}_{2}\left(x,y,z,t\right)\right)}^{2}$$

39 $${G}_{V}\left(x,y,z,t\right)=\frac{{\varGamma}_{1}\left(x,y,z,t\right){e}^{\delta1\left(x,y,z,t\right)}+{\varGamma}_{2}\left(x,y,z,t\right){\delta}_{2}\left(x,y,z,t\right)}{{\omega}_{51}{e}^{\delta1\left(x,y,z,t\right)}+{\omega}_{52}{\left(\delta2\left(x,y,z,t\right)\right)}^{2}}$$

where

40 $$ \begin{gathered} \Gamma _{1} \left( {x,y,z,t} \right) = 2\omega _{{51}} \left( { - \eta _{{11}} o_{{11}} \sin \left( {\xi _{{15}} \left( {x,y,z,t} \right)} \right) + \eta _{{12}} o_{{21}} \cos \left( {\xi _{{25}} \left( {x,y,z,t} \right)} \right)} \right) \hfill \\ \Gamma _{2} \left( {x,y,z,t} \right) = 4\omega _{{52}} \left( { - \eta _{{21}} o_{{21}} \sin \left( {\xi _{{25}} \left( {x,y,z,t} \right)} \right) + \eta _{{22}} o_{{11}} \cos \left( {\xi _{{15}} \left( {x,y,z,t} \right)} \right)} \right) \hfill \\ \end{gathered} $$

The set of coefficients corresponds to this case is,

41 $$SetV=\left\{{o}_{12}={o}_{13}={o}_{21}={v}_{52}={\eta}_{11}={\eta}_{21}=0,{v}_{51}=\frac{{o}_{11}(8{o}_{11}^{2}{o}_{22}+3{o}_{23})}{2{o}_{22}}\right\}$$

The solution function ${G}_{V}\left(x,y,z,t\right)$ combines oscillatory terms (cosine and sine functions) and exponential growth in a more complex, coupled manner. The numerator is composed of two terms: the first includes an exponential factor modulated by ${\delta}_{1}(x,y,z,t)$ and ${\varGamma}_{1}(x,y,z,t)$ that lead to oscillatory terms, while the second term features mixed trigonometric components of ${\delta}_{2}(x,y,z,t)$ and ${\varGamma}_{2}(x,y,z,t)$, both contributing to the overall behavior. The denominator combines exponential and quadratic terms, creating a nonlinear structure that involves both growing and decaying components. These components together form a function with significant dynamical complexity, driven by the interplay between spatial oscillations and temporal growth.

To simplify the system and explore its behavior under specific conditions, we consider the parameter set Set $V$, where many of the cross-coupling terms are set to zero. In this set, ${o}_{12}={o}_{13}={o}_{21}={v}_{52}={\eta}_{11}={\eta}_{21}=0$, and the velocity term ${v}_{51}$ is defined as

42 $${v_{51}}=\frac{{{o_{11}}\left( {8o_{{11}}^{2}{o_{22}}+3{o_{23}}} \right)}}{{2{o_{22}}}}$$

which creates a specialized relationship between the parameters. This reduction significantly simplifies the structure of the function, focusing its behavior on the interaction between the terms ${\delta}_{1}$ and ${\delta}_{2}$ in the numerator and denominator, allowing for a clearer examination of the underlying dynamics.

Overall, the function ${G}_{V}\left(x,y,z,t\right)$ represents a nonlinear wave phenomenon characterized by the interaction of sinusoidal and exponential components. The specific parameterization in Set V provides a clear example of how the function can be simplified to model localized excitations, with significant contributions from both oscillatory behavior and exponential growth. This function could be relevant for describing complex resonant systems where different spatial-temporal modulations interact to produce rich nonlinear dynamics, including both local resonances and oscillatory wave patterns.

### Graphical analysis of some obtained solutions

In this section, a visual analysis has been provided of the exact solutions derived earlier to grasp the dynamical behaviors of the (3 + 1)-dimensional Jimbo-Miwa equation (JME). While algebraic expressions offer precision, graphical representations are essential for interpreting complex physical features, such as steep localized transitions and nonlinear wave interactions, which are often difficult to discern from equations alone.

Using the developed bilinear neural network architectures, ranging from 4-2-1 to 4-4-1 model, we have generated 3D surface structures, density contour plots, and 2D cross-sectional profiles for some obtained solutions. These visualizations, constructed using specific parameter sets, validate the framework’s ability to capture intricate nonlinear dynamics and provide deeper insight into the stability and propagation characteristics of these high-dimensional waves.

**Fig. 5:**
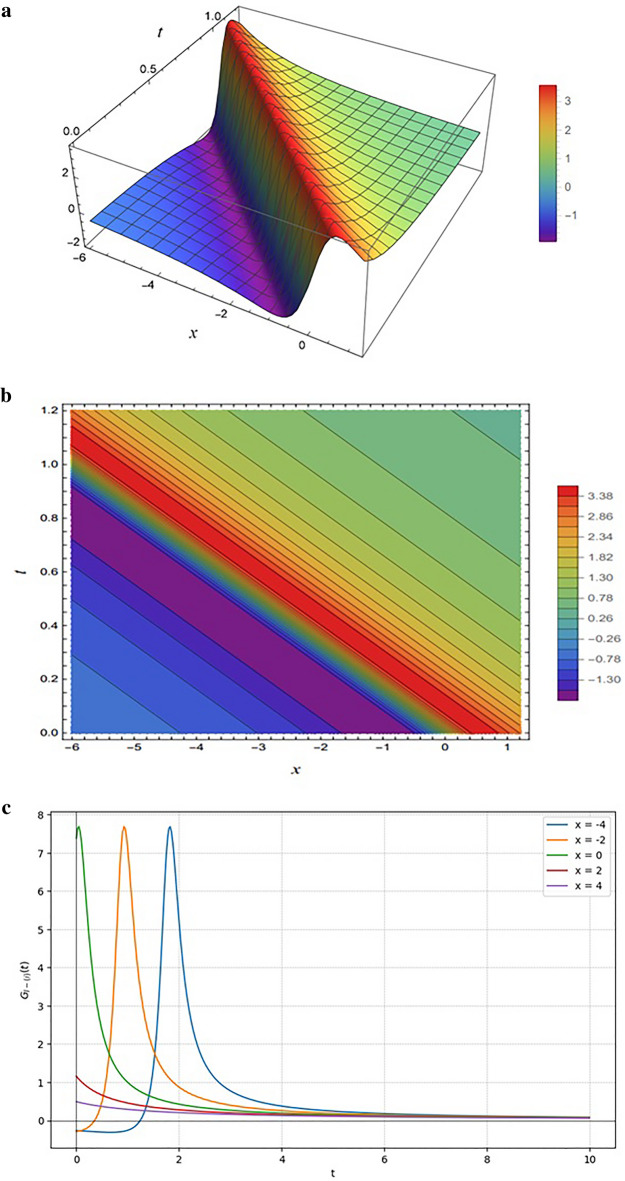
Representations of the solution ${G}_{I-\left(1\right)}(x,y,z,t)$ given by Eq. (13): (**a**) 3D surface plot, (**b**) density plot, and (**c**) 2D profile plots. All results are generated using the input functions defined in Eq. (9) and the parameter set specified in Eq. (11).

Figure 5 displays the solution ${G}_{I-\left(1\right)}$, obtained from Eq. (11) under the parameter choice $SetI-1$. The function is governed by a rational form where the numerator contains linear combinations of the phase variables ${\xi}_{11}(x,y,z,t)$ and ${\xi}_{21}(x,y,z,t)$, while the denominator involves quadratic contributions of the same terms.

Figures 5(a) and 5(b) illustrate the solution’s spatial-temporal structure, where the numerator’s linear dependence creates a sharp ridge and dense diagonal contours, while the quadratic denominator ensures smooth decay and stabilization away from this central region. This interplay links the algebraic form to the physical profile, which Fig. 5c identifies as a localized “lump solution” that maintains a stable amplitude while its peak position propagates along the $t$-axis as the spatial coordinate $x$ varies.

**Fig. 6:**
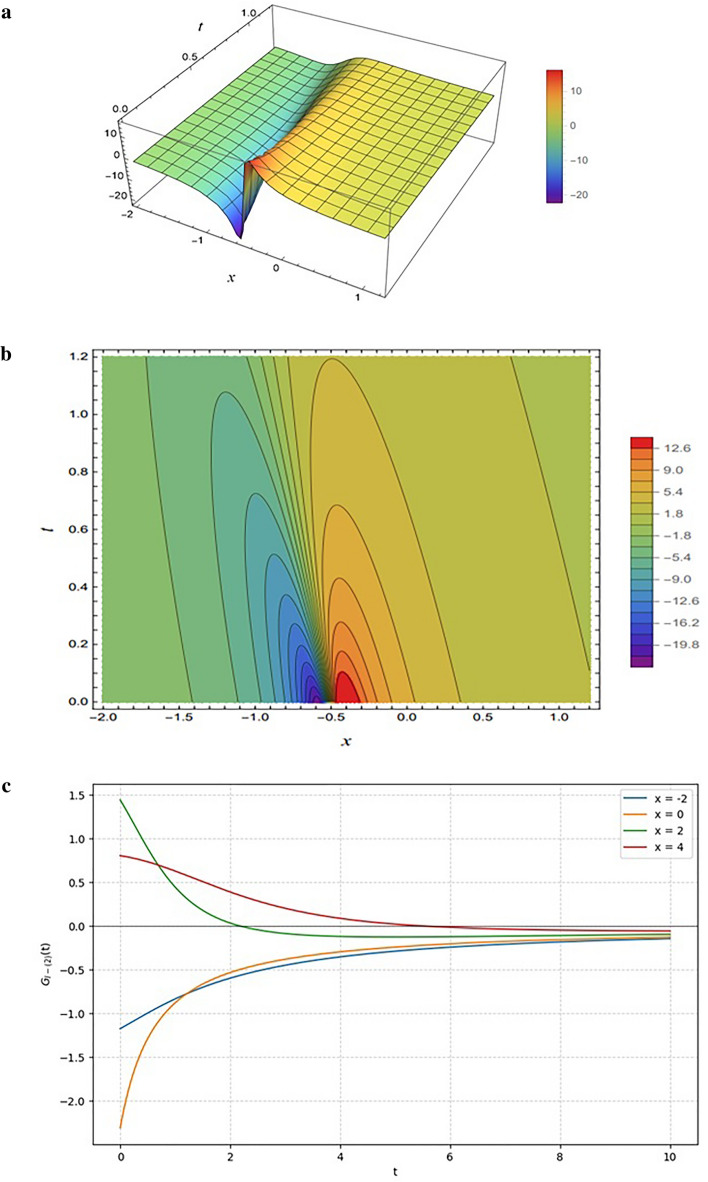
Same as Fig. 5 but generated using the parameter set specified in Eq. (12).

Figure 6 illustrates the transient solution ${G}_{I-\left(2\right),}$, characterized by a sharp valley-ridge profile in the 3D plot (Fig. 6a) and dense, curved gradients in the contour plot (Fig. 6b) that arise from dominant linear numerator terms. The contour plot illustrates that the clustering of contour lines near the origin marks rapid changes introduced by the numerator’s terms, while the stretched lines farther out reflect the stabilizing role of the denominator. In contrast to the stable propagating lump observed previously, the 2D profiles in Fig. 6c demonstrate asymptotic decay, where the wave amplitude rapidly relaxes toward a zero-equilibrium state as $t$ increases, indicating that the solution dissipates over time rather than maintaining a sustained pulse.

Figure 6 shows the solution ${G}_{I-\left(2\right),}$ obtained from Eq. (13) with the parameter choice ($SetI-2$)$.$The function maintains the same rational structure as in the previous case, but the modified parameter set changes the behavior of the solution.

**Fig. 7:**
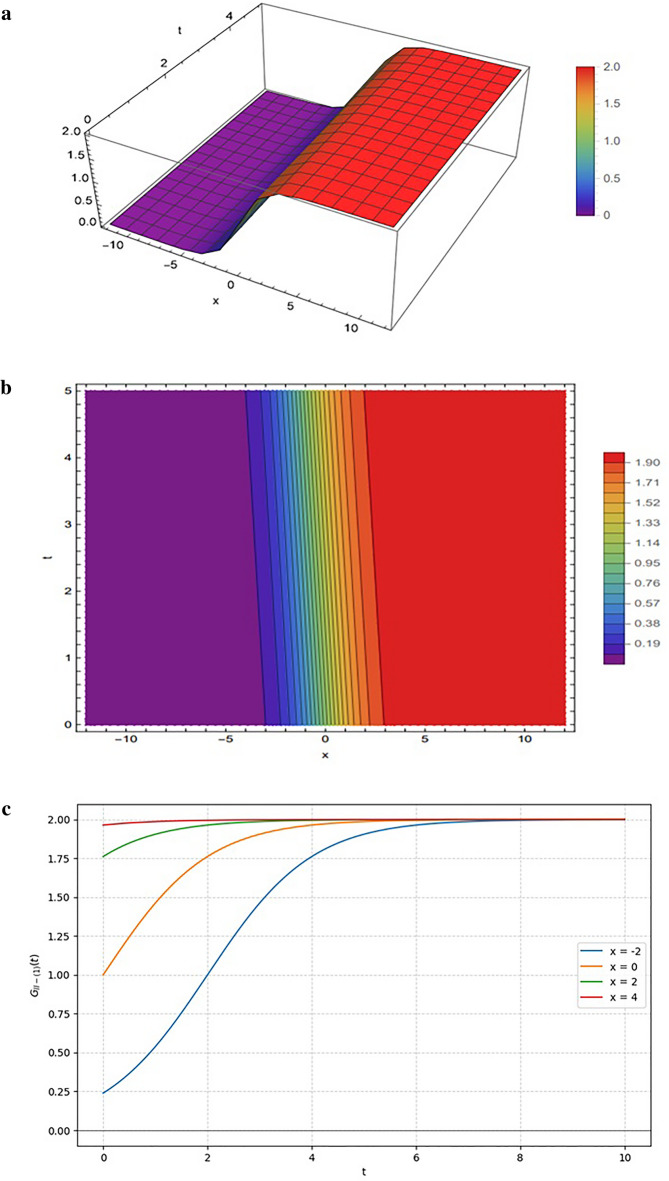
Representations of the solution ${G}_{II-\left(1\right)}(x,y,z,t)$ given by Eq. (20): (**a**) 3D surface plot, (**b**) density plot, and (**c**) 2D profile plots. All results are generated using the input functions defined in Eq. (14) and the parameter set specified in Eq. (16).

Figure 7 depicts the solution ${G}_{II-\left(1\right)}$, generated from the functional form in Eq. (20) under the parameter choice $SetII-1$. The structure of the function combines a linear contribution in ${\xi}_{12}(x,y,z,t)$ with an exponential contribution in ${\xi}_{22}(x,y,z,t)$, balanced by quadratic and exponential terms in the denominator.

Figure 7 illustrates the emergence of a topological solitary wave (kink) resulting from the parameter condition ${{\upomega}}_{12}=0$ in Eq. (16), which eliminates the rational component of the solution. The 3D surface plot in Fig. 7a and the density plot in Fig. 7b characterize this as a smooth step-like transition, where the exponential growth is saturated by the denominator to create distinct, flat plateaus separated by a region of rapid gradient change. This behavior is further detailed in the 2D propagation profiles of Fig. 7c, which exhibit a characteristic sigmoidal evolution; unlike the transient, decaying lump solutions observed in Case I, these curves evolve monotonically and stabilize at a non-zero constant (approximately 2.0), confirming the solution behaves as a stable transition between equilibrium states.

**Fig. 8:**
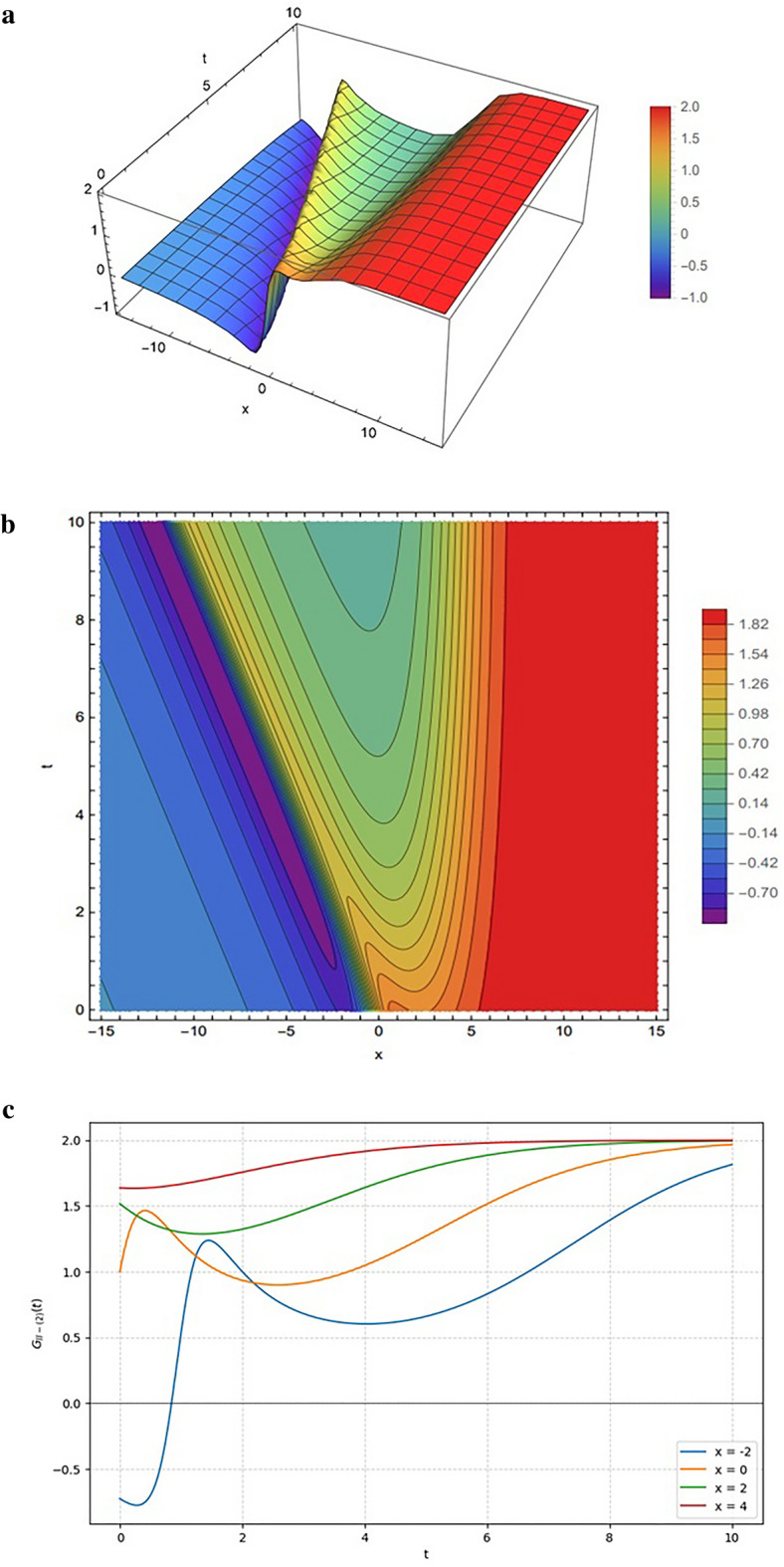
Same as Fig. 7 but generated using the parameter set specified in Eq. (17).

Figure 8 shows the solution ${G}_{II-\left(2\right)}$ (Eq. (20) using the parameter $setII-2$. The functional form combines linear contributions in ${\xi}_{12}(x,y,z,t)$ with exponential terms in ${\xi}_{22}(x,y,z,t)$, moderated by quadratic and exponential terms in the denominator. This structure produces a mixed profile of oscillatory and step-like behavior.

Figure 8 illustrates the hybrid interaction dynamics of the solution ${G}_{II-\left(1\right)}$ (Eq. 20) under the parameter set in Eq. (17). Figure 8a highlights this interaction in the 3D surface plot, showing initial oscillatory troughs and peaks driven by the numerator’s linear-exponential interplay before smoothing into a stable plateau. This transition is reinforced by Fig. 8b, where densely packed contours mark the region of rapid change while broad spacing indicates stabilization. Figure 8c quantifies these dynamics, revealing distinct non-monotonic fluctuations-such as the sharp dip at $x=-2$ caused by the rational lump component before the exponential term dominates and saturates the amplitude at a stable equilibrium of approximately 2.0.

**Fig. 9:**
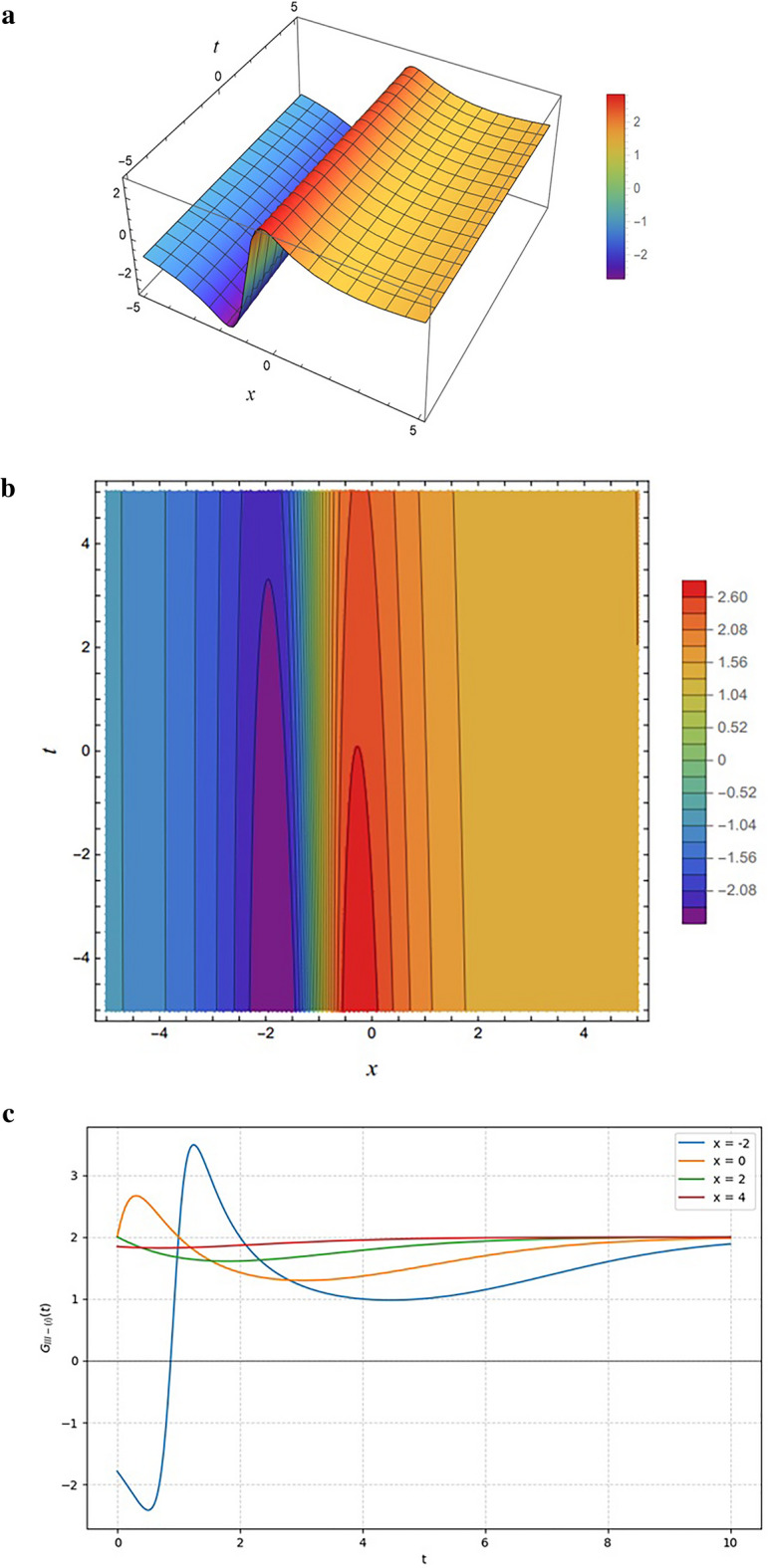
Representations of the solution ${G}_{III-\left(1\right)}(x,y,z,t)$ given by Eq. (23): (**a**) 3D surface plot, (**b**) density plot, and (**c**) 2D profile plots. All results are generated using the input functions defined in Eq. (21) and the parameter set specified in Eq. (24).

Figure 9 displays the solution ${G}_{III-\left(1\right)}$, obtained from Eq. (23) under the parameter selection $SetIII-1$ (Eq. (24)). The structure of the generating function incorporates linear contributions through ${\xi}_{13}(x,y,z,t)$ and ${\xi}_{23}(x,y,z,t)$, combined with exponential contributions from ${\xi}_{33}(x,y,z,t)$ and ${\xi}_{43}(x,y,z,t)$. The denominator contains quadratic and exponential terms, which act to regulate the solution’s growth and maintain stability.

**Fig. 10:**
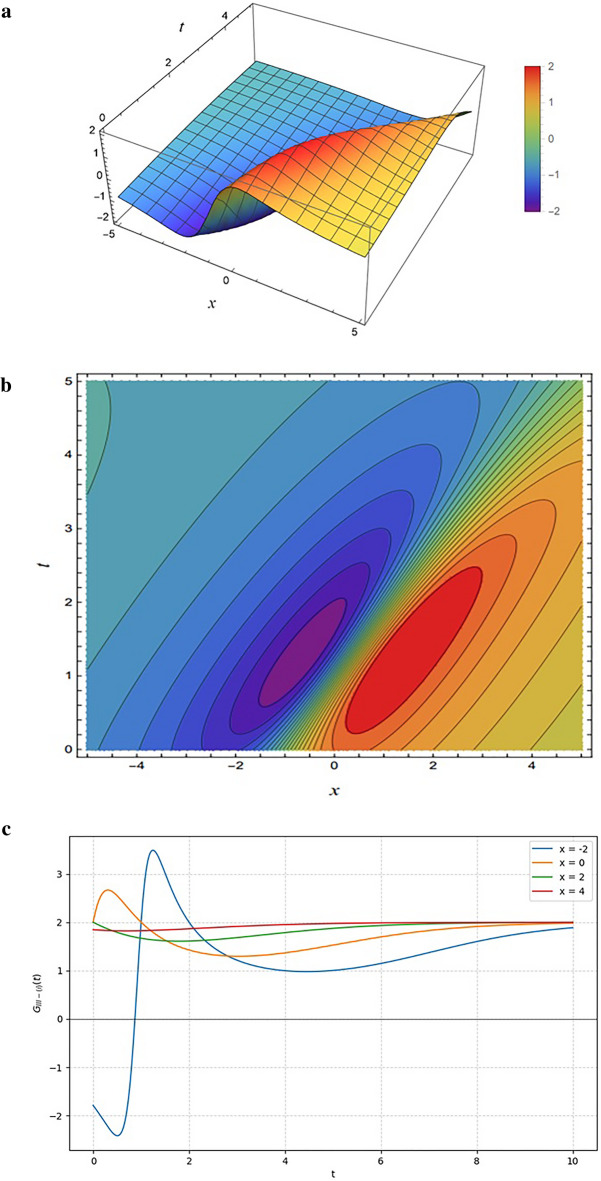
Representations of the solution ${G}_{III-\left(3\right)}(x,y,z,t)$ given by Eq. (23): (**a**) 3D surface plot, (**b**) density plot, and (**c**) 2D profile plots. All results are generated using the input functions defined in Eq. (21) and the parameter set specified in Eq. (26).

Figure 9 illustrates the higher-order interaction dynamics governed by Eq. (23) and the parameter set in Eq. (24). Figure 9a displays a sharp transition region where the solution rises steeply due to dominant exponential terms in the numerator, while Fig. 9b highlights this gradient through vertical layers and symmetric central pockets that reflect the interacting exponential modes. In contrast to smoother previous cases, the 2D temporal evolution in Fig. 9c reveals a high-amplitude resonant interaction; specifically, the profile at $x=-2$ undergoes a violent transient oscillation-dropping to approximately − 2.4 before surging to a peak of 3.5 -indicating a strong collision between the rational lump and exponential wavefronts that eventually stabilizes into a topological kink structure.

Figure 10 illustrates the solution ${G}_{III-\left(3\right)}$, obtained from Eq. (23) under the parameter selection $SetIII-3$ (Eq. (26)). The generating function integrates linear contributions through ${\xi}_{13}(x,y,z,t)$ and ${\xi}_{23}(x,y,z,t)$, together with exponential contributions from ${\xi}_{33}(x,y,z,t)$ and ${\xi}_{43}(x,y,z,t)$. The denominator incorporates quadratic and exponential terms, which play a stabilizing role in balancing the sharp variations in the numerator and ensuring the controlled evolution of the solution.

Figure 10 illustrates the solution ${G}_{III-\left(3\right)}$ derived from Eq. (23) using the parameter set in Eq. (26), where the interaction of linear and exponential numerator terms is balanced by a stabilizing quadratic and exponential denominator. The 3D surface plot in Fig. 10a displays a steep, smooth transition marking a localized wavefront dominated by exponential growth, while the contour plot in Fig. 10b highlights the resulting sharp variation zones through localized lobes and layered gradients. These high-order interaction dynamics are quantified in Fig. 10(c), which reveals a robust resonant collision; specifically, the profile at $x=-2$ exhibits a violent transient fluctuation-dropping to a rarefaction of $\approx-2.4$ followed immediately by a compression peak of $\approx3.5$ in the interval $t\in\left[\mathrm{0,2}\right]$-before the oscillations damp out and the system converges asymptotically to a stable equilibrium state of $\approx2.0$.

**Fig. 11:**
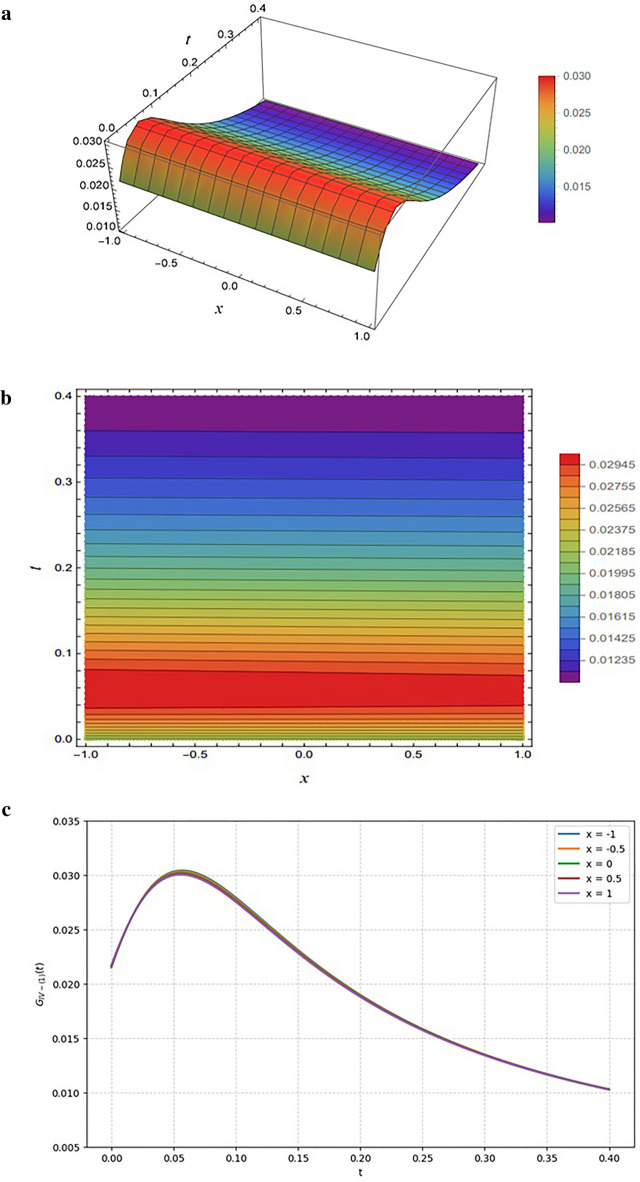
Representations of the solution ${G}_{IV-\left(1\right)}(x,y,z,t)$ given by Eq. (31): (**a**) 3D surface plot, (**b**) density plot, and (**c**) 2D profile plots. All results are generated using the input functions defined in Eq. (29) and the parameter set specified in Eq. (32).

Figure 11 illustrates the solution ${G}_{IV-\left(1\right)}$, obtained from Eq. (31) under the parameter selection $SetIV-1$ (Eq. (32)). The generating function structure involves hyperbolic and trigonometric contributions, with terms such as $cosh\left({\xi}_{14}(x,y,z,t)\right),sinh\left({\xi}_{14}(x,y,z,t)\right)$, and $cos\left({\xi}_{34}(x,y,z,t)\right)$, combined with polynomial contributions through ${\xi}_{24}(x,y,z,t)$. The denominator incorporates quadratic and trigonometric-hyperbolic terms, which regulate oscillations and maintain the boundedness of the solution.

Figure 11 illustrates the solution ${G}_{IV-\left(1\right)}$ defined by the mixed hyperbolic-rational trigonometric structure in Eq. (31) using parameter Set IV-1 (Eq. 32). The 3D surface plot in Fig. 11a demonstrates a stable solution characterized by a nearly flat plateau, indicating that the denominator significantly dampens the oscillatory trigonometric terms. This is reinforced by the contour plot in Fig. 11b, where horizontal bands reveal weak oscillatory modes and a layered structure, confirming the generation of controlled oscillations without divergence. Figure 11c details the 2D temporal evolution, which generates a single, temporally localized pulse; specifically, the amplitude rises rapidly to a peak of approximately 0.03 at $t\approx0.06$ before undergoing smooth asymptotic decay due to stabilizing hyperbolic sine and quadratic contributions. The nearly identical trajectories across varying spatial coordinates $(x\in[-\mathrm{1,1}\left]\right)$ suggest a uniform “breather-like” excitation where temporal dynamics dominate spatial dispersion.

**Fig. 12:**
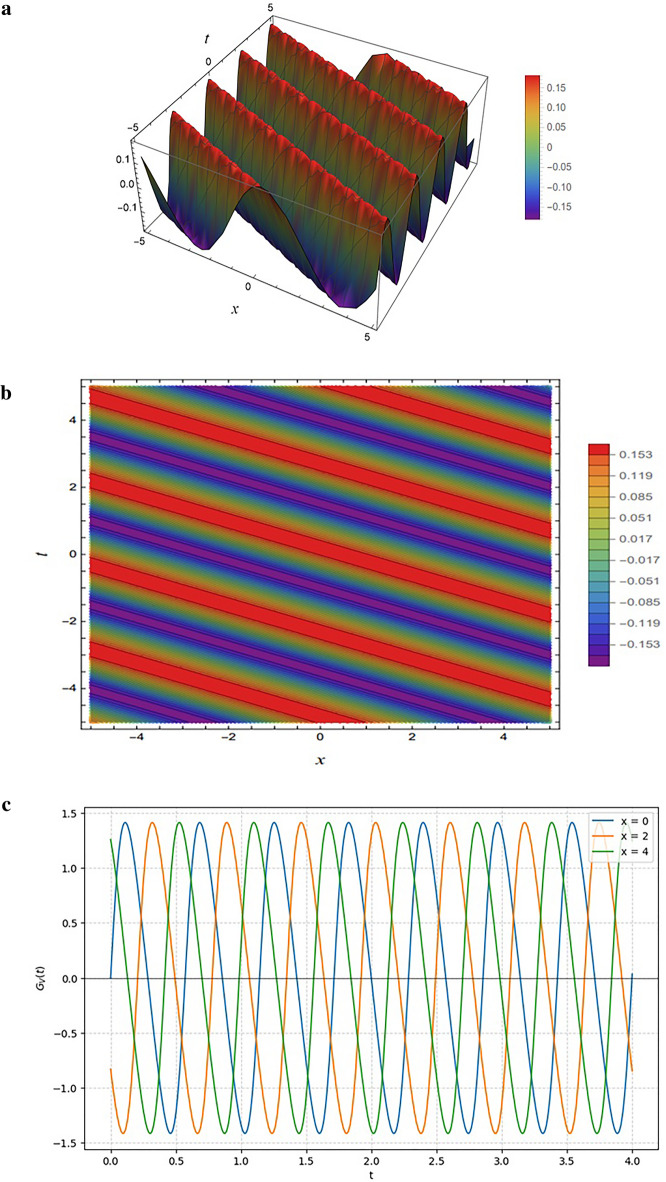
Representations of the solution ${G}_{V}(x,y,z,t)$ given by Eq. (39): (**a**) 3D surface plot, (**b**) density plot, and (**c**) 2D profile plots. All results are generated using the input functions defined in Eqs. (36) and (37) and the parameter set specified in Eq. (41).

Figure 12 illustrates the solution ${G}_{V}(x,y,z,t)$, obtained from Eq. (39) under the parameter selection $SetV$. The structure of the generating function is defined by the exponential and trigonometric components ${\xi}_{15}(x,y,z,t)$ and ${\xi}_{25}(x,y,z,t)$, which feed into the nonlinear oscillatory terms ${\delta}_{1}(x,y,z,t)$ and ${\delta}_{2}(x,y,z,t)$. The numerator involves ${\varGamma}_{1}(x,y,z,t)$ and ${\varGamma}_{2}(x,y,z,t)$, both of which combine sinusoidal and cosinusoidal interactions with the parameters ${\eta}_{ij}$ and ${o}_{ij}$, leading to oscillatory contributions. The denominator contains exponential and quadratic terms, ensuring that the oscillations are modulated and bounded.

Figure 12 illustrates the periodic solution ${G}_{V}$ derived from the complex rational-exponentialtrigonometric structure in Eq. (39) using parameter Set V (Eq. 41). Figure 12a reveals a highly oscillatory 3D surface with alternating peaks and troughs, driven by the trigonometric coupling of ${\delta}_{1}$ and ${\delta}_{2}$ (Eq. 37) and reinforced by the exponential denominator. This periodic nature is emphasized in Fig. 12b, where diagonal bands of alternating colors highlight repeating constructive and destructive interference patterns. In sharp contrast to previous decaying or saturating cases, Fig. 12c demonstrates a sustained periodic wave train; the amplitude fluctuates symmetrically between approximately − 1.4 and + 1.4 without attenuation, and the phase shifts between spatial curves $(x=\mathrm{0,2},4)$ confirm the solution behaves as a stable, non-dissipative traveling wave.

## Conclusion

In this work, we investigated the ( $3+1$ )-dimensional Jimbo-Miwa equation (JME), an important integrable model within the KP hierarchy that captures nonlinear wave dynamics in plasma physics and nonlinear optics. By applying the Cole-Hopf transformation, we derived its bilinear form and systematically constructed a wide variety of exact solutions, including lump solutions, lump-soliton interactions, lump-double soliton interactions, breather solutions, and mixed resonant structures generated through different neural network architectures.

The results demonstrate that the JME admits a remarkably rich solution space characterized by diverse nonlinear behaviors. Lump-type solutions highlight localized structures governed by rational forms, while lump soliton and lump double soliton interactions reveal the complexity of resonant propagation and asymmetric wavefronts. Breather-type solutions further extend this variety, showcasing oscillatory and exponentially modulated dynamics with potential relevance for physical systems such as supercontinuum generation and optical frequency combs. The constructed “4-2-2-1” neural network model additionally illustrates how oscillatory and exponential contributions can be coupled to describe hybrid resonant patterns, thereby providing deeper insight into the interplay of polynomial, trigonometric, hyperbolic, and exponential modes.

Overall, our findings enrich the understanding of the Jimbo-Miwa equation by not only confirming its integrability through bilinearization but also by providing a unified framework to generate and interpret a broad spectrum of nonlinear wave phenomena. These results emphasize the versatility of bilinear and neural-network-inspired approaches in uncovering exact solutions for high-dimensional nonlinear systems. Future work may explore the stability, perturbation dynamics, and physical realizations of these solutions in experimental settings, further bridging the gap between mathematical theory and practical applications.

As natural extensions, future work aims to enrich the framework by introducing stability analysis to verify physical robustness against perturbations, while multistability analysis could reveal the coexistence of multiple stable wave states under specific parametric conditions^21^.

## Data Availability

All data generated or analyzed during this study are included in this published article.
